# Unexpected Fascicle Length Changes In Denervated Feline Soleus Muscle During Stance Phase Of Walking

**DOI:** 10.1038/srep17619

**Published:** 2015-12-04

**Authors:** Ricky Mehta, Huub Maas, Robert J. Gregor, Boris I. Prilutsky

**Affiliations:** 1 School of Applied Physiology, Center for Human Movement Studies, Georgia Institute of Technology, Atlanta, GA, USA; 2 Department of Human Movement Sciences, Faculty of Behaviour and Movement Sciences, Vrije Universiteit Amsterdam, MOVE Research Institute Amsterdam, The Netherlands; 3 Division of Biokinesiology and Physical Therapy, University of Southern California, Los Angeles, CA, USA

## Abstract

After surgical repair of traumatically severed peripheral nerves, associated muscles are paralyzed for weeks. Little is known about fascicle length changes in paralyzed muscles during locomotion. The aim of this study was to investigate to what extent, if any, muscle fascicles of denervated feline soleus (SO) change length during stance of walking when intact SO synergists are actively contracting. Hindlimb kinematics, SO fascicle and muscle-tendon unit (MTU) length, and EMG activity of SO, lateral gastrocnemius (LG) and medial gastrocnemius (MG) were measured during level and slope walking in adult cats. Measurements were taken before and 1–2 weeks following SO-LG denervation. Unexpectedly, SO fascicle lengthening and shortening during stance in all walking conditions were evident after denervation. The greatest SO fascicle shortening (17.3 ± 2.2% of a reference length) and least fascicle lengthening (1.5 ± 0.8%) after denervation were found during upslope walking, where MG EMG activity was greatest across slopes (P < 0.05) and greatest discrepancies between post denervation SO fascicle and MTU length changes occurred. These findings suggest that myofascial linkages between denervated SO and its active synergists might affect its fascicle length changes. Further studies are needed to directly test this suggestion.

Peripheral nerve injury causes long-lasting and often permanent sensorimotor deficits even after the severed nerve is surgically repaired^1^,^2^,^3^,^4^. After surgical repair of a severed peripheral nerve, the muscle remains in a state of paralysis while motor and sensory axons from the proximal nerve stump are regenerating at a rate of 1–3 mm/day to reach the target muscle^5^,^6^. During this time, the paralyzed muscle is prone to atrophy^7^,^8^. Permanent or long-lasting atrophy can reduce the magnitude and symmetry of loading of the joint and cause subsequent development of joint tissue degeneration, osteoarthritis, and other secondary joint conditions^9^,^10^,^11^. Paralysis of selected ankle extensors in animal models changes inter-joint coordination as well as activity and fascicle length changes in intact synergists^12^,^13^,^14^,^15^.

Although there are indications of mechanical interactions between passive muscle heads of the human triceps surae *in vivo*^16^,^17^ and between muscles in the injured human hamstrings group after rupture of synergist tendon and muscle retraction^18^, detailed information about the *in vivo* mechanical conditions of muscle fascicles in a denervated muscle surrounded by intact synergists is currently unavailable. The extent to which fascicles of a denervated muscle passively lengthen and shorten while its intact synergists undergo active lengthening and shortening during normal motor behaviors is unknown. Information about muscle fascicle mechanical behavior in the injured muscle, specifically in denervated muscle, would improve our understanding of the mechanical behavior of paralyzed muscles following peripheral nerve injury.

The aim of this study was to investigate to what extent, if any, muscle fascicles of the denervated feline SO change length during the stance phase of walking. In a denervated muscle, there is no active force generation, whereas passive force production by cat SO muscle-tendon unit (MTU) stretched by ankle dorsiflexion up to ~90^o^–120^o^ (angles corresponding to the maximum ankle dorsiflexion in the stance phase of cat level and slope ( ± 50% or  ± 27^o^) walking^19^) is negligible^20^,^21^. Fascicles of the passive cat SO have a greater compliance compared to the tendon^22^. Finally, a negligible amount of myofascial force transmission has been reported between SO and its synergists^23^,^24^. Based on the above observations, we expected that SO fascicles would mostly mirror lengthening of the MTU during ankle yield in the stance phase of walking. During passive ankle extension (plantarflexion) caused by external forces applied to the foot while all ankle muscles are relaxed, the fascicles and tendon of denervated SO would be expected to buckle as shown to be the case at short lengths of passive muscles *in vivo*^25^,^26^. On the other hand, we expected little if any buckling or shortening of denervated SO fascicles during ankle extension in stance of walking. Buckling of passive SO fascicles during SO MTU shortening would be prevented by high pressure inside the superficial posterior crural compartment^27^,^28^ developed by bulging fascicles of active muscles^29^ surrounding SO. Substantial shortening of the denervated SO fascicles would be also unlikely because of limited myofascial force transmission from contracting synergists^23^,^24^.

Preliminary results of this study have been published in abstract form^30^.

## Methods

### Ethical approval and animal training

The surgical and experimental procedures employed corresponded to the “Principles of Laboratory Animal Care” (NIH Publication No. 86–23, Revised 1985) and were approved by the Institutional Animal Care and Use Committee of the Georgia Institute of Technology. Seven adult female cats (*Felis domesticus*, mass 3.7 ± 0.8 kg; mean ± SD) were studied. Cats were trained using operant conditioning methods to walk within a custom-made, Plexiglas-enclosed walkway (3.0 m × 0.4 m) with up to three embedded three force platforms (16 cm × 11 cm and 11 cm × 7 cm; Bertec Corporation, Columbus, OH, USA) and covered with nonslip rubberized matting. Cats were trained to walk at three walkway slopes – level surface (0%, i.e. 0^o^), upslope (50%, i.e. 27^o^) and downslope (−50%, i.e. −27°) – for several hours a day, 5 days a week for 3–4 weeks; for more detail see^19^,^31^.

### Surgical procedures

The surgical procedures have been described in detail previously^14^,^19^,^32^ and, therefore, only a brief account of the procedures is provided below. Following locomotor training and initial locomotor recordings, each cat underwent two survival surgeries under aseptic conditions and general isoflurane anesthesia.

### Implantation surgery

The animal was anesthetized using ketamine (10 mg/kg, S.C.), atropine (0.05 mg/kg, S.C.) and isoflurane (inhalation, 5%) and anesthesia was maintained with isoflurane (1–3%). The animal’s vital physiological parameters (temperature, respiration, heart rate, blood pressure) were monitored throughout the surgery. After shaving and cleaning with surgical disinfectant the skin overlying the skull, the low back, and dorsal aspects of the right shank, skin incisions were made. Teflon-insulated multi-stranded stainless steel fine wires (CW5402; Cooner Wire, Chatsworth, CA, USA) and leads with piezoelectric sonomicrometry crystals (2 mm in diameter; Sonometrics, London, ON, Canada) were passed subcutaneously along the back from the skull to an incision in the hindlimb. Four of the seven cats were implanted with the sonomicrometry crystals. All wire leads were attached to two multi-pin Amphenol connectors that were secured to the skull with four stainless steel or titanium screws and dental cement. A thin strip of insulation (~1 mm) was removed near the distal end of each pair of the fine wires and secured inside the muscle belly of SO, LG and MG muscles. Mild electrical stimulation of the muscles through the head connectors was used to verify wire placements. The implanted fine wires were used to record electromyographic (EMG) activity. To measure SO fascicle length, one pair of the piezoelectric crystals was implanted near the origin and insertion (i.e., proximal and distal aponeurosis) of SO muscle fascicles along the muscle belly midline. The superficial surface of the muscle belly at the location of the crystal implantation was reached through a small cut of the fascia surrounding the muscle. A small pocket inside the muscle was created between the muscle fibers. The crystal was inserted in the pocket beneath the muscle surface, and the pocket was closed by a 4–0 silk suture, which also anchored the crystal lead wire.

After implantation of EMG electrodes and sonomicrometry crystals, the skin incisions were closed using Vicryl 4–0 suture for the deep fascia and Vicryl 5–0 subcuticular suture for dermal closure. The animal recovered after surgery for 14 days with pain medication administered for at least 3 days and antibiotics for 10 days.

After animal recovery, hindlimb mechanics, EMG activity of SO, LG, and MG muscles, and SO muscle fascicle lengths were collected during level, downslope and upslope walking (see below) to obtain baseline values.

### Nerve transection and repair surgery

These surgical procedures were similar to those described previously^13^,^14^,^33^. Animal preparation, anesthesia, monitoring during surgery and pain medication after surgery were the same as described for the implantation surgery. The branch of the tibial nerve supplying SO and LG muscles was reached through a longitudinal incision in the popliteal region of the right hindlimb. A small portion of the nerve branch (~2–4 mm) was carefully separated from surrounding tissues leaving the fat pad in the popliteal region intact and then transected with sharp scissors. Muscle denervation was verified by mild electrical stimulation of the proximal nerve stump. The transected nerve stumps were approximately aligned with each other and secured in place by fibrin glue (equal parts of thrombin and a 1:1 mixture of fibrin and fibronectin; Sigma-Aldrich, St. Louis, MO, USA). After the surgery the animal recovered for 3–5 days before the locomotion experiments resumed.

### Locomotion Experiments

Each cat was tested in two series of locomotion experiments before and after SO-LG nerve transection and repair to measure hindlimb mechanics, EMG of SO and MG, as well as SO fascicle length. Locomotor data collection before nerve injury was performed approximately 2 hours a day, 3–5 days a week for about a month. Post nerve injury locomotor data reported here were collected during the first 3–14 days (1–2 weeks) after SO-LG nerve transection and repair using the same recording schedule as for pre-nerve injury data collection.

In order to record hindlimb kinematics, light reflective markers were placed on the following right hindlimb anatomical landmarks using double-sided adhesive tape: iliac spine, greater trochanter, lateral femoral epicondyle, lateral malleolus, 5th metatarsophalangeal (MTP) joint and the distal end of the 5th digit. Marker positions were recorded using a 3D, 6-camera motion capture system (Vicon Motion Systems Ltd, Oxford, UK) at a sampling rate of 120 Hz. The three components of the ground reaction force vector and coordinates of its application point to the paw were recorded at a sampling rate of 360 Hz by 1 of the 3 small force plates (Bertec Corporation, Columbus, OH, USA) embedded into the walkway surface and actually contacted during any specific trial. SO fascicle length was recorded by the sonomicrometry system (Sonometrics Corporation, London, ON, Canada) at a sampling rate of 1059 Hz. EMG signals were collected at a sampling rate of 3000 Hz, band-pass filtered (30–1000 Hz, 3 dB), amplified (100×), and saved on a PC for further analysis. The EMG and sonomicrometry signals were collected via a 16-conductor shielded flexible cable attached to the Amphenol connectors on the animal’s head. The simultaneous data collection onset was triggered by the electronic pulse of the Vicon system to synchronize recordings of the walking mechanics, EMG and SO fascicle length.

### Data analysis

*Hindlimb kinematics*. Recorded coordinates of markers on the right hindlimb were low-pass filtered using a Butterworth low-pass, zero-lag filter (cut-off frequency 6 Hz). To reduce knee marker errors due to excessive skin movement, the estimated knee joint center position at each video frame was recalculated using the recorded position of the ankle and hip joints and length of the shank and thigh segments^34^; the segment lengths were measured by a caliper during the surgery. Individual walking cycles and their stance and swing phases were identified as the periods between the right hind paw contact (PC) and paw-off (PO) time instances determined from the force plate recordings. The computed hindlimb joint angles as defined in^19^ and a geometric musculoskeletal model of the hindlimb^34^ were used to calculate SO muscle-tendon unit (MTU) length. MTU length was normalized to a reference length (L_ref_) defined as the mean between the maximum and minimum of the averaged MTU lengths during the swing phase in baseline level walking^32^.

*EMG*. Recorded EMG signals were band-pass filtered (see above) and full-wave rectified. Within each walking cycle an EMG burst of SO, LG or MG was considered on when the EMG signal was above a threshold signal level for at least 50 ms, and off when the signal was below the threshold for at least 50 ms. The threshold level was defined as 2 s.d. above the mean EMG signal during a muscle silent period (during most of the swing phase of walking)^19^. Mean EMG burst activity for each of the three muscles was determined as the ratio of the time integral of the rectified EMG burst and the burst duration. The mean EMG burst magnitudes of each muscle within an animal were normalized by the maximum mean burst magnitude found across all walking cycles and conditions recorded before denervation.

*SO fascicle length*. Fascicle length was computed from the measured propagation time of ultrasound between the implanted piezoelectric crystals and the known speed of ultrasound propagation in muscle tissue (1540 m/s^35^). Fascicle length calculations and low-pass filtering was done using the SonoView software (Sonometrics Corporation, London, ON, Canada). The SO fascicle length was normalized to a reference length defined as the mean of the maximum and minimum fascicle lengths during the swing phase of level walking^32^.

### Fascicle length measurements in a sedated cat

To assess the minimum SO fascicle length in relaxed muscle, fascicle length measurements were made in one cat (CO) before SO-LG denervation while the cat was sedated (dexmedetomidine, 40–60 μg/kg, S.C.). The ankle joint angle was fully plantar flexed at 180^o^ and the knee joint flexed at 35^o^. SO fascicle length was also measured at the same ankle and knee joint angles while the SO was electrically stimulated through the implanted EMG electrodes. A single pulse (100 ms) was used with the current selected to cause maximum fascicle shortening.

### Treatment of data and statistics

Walking cycles with a steady-state speed and without paw slippage were selected and used for further analysis. For MTU and muscle fascicle length analysis, a total of 186 walking cycles were processed across 4 cats with implanted sonomicrometry crystals, pre and post SO-LG denervation, and three walking slope conditions (Table 1). For EMG analysis, 289 walking cycles across 7 cats with implanted EMG electrodes (including 4 cats with sonomicrometry crystals) and all experimental conditions were processed. In some cats, we were unable to analyze EMG data due to the low signal-to-noise ratio, specifically for MG in downslope and level walking before denervation. This could have resulted from implantation of electrodes in the mid-belly compartment of MG with a low percentage of slow-twitch fibers (A. W. English, personal communication). As a result, relatively low EMG activity could be detected during downslope and level walking at self-selected speeds, as opposed to upslope walking that requires recruitment of additional faster motor unit populations^19^,^36^. EMG activity of MG also increases following denervation of its synergists^13^,^14^,^37^. For detailed information about the number of trials included in our analysis, see Table 1. For the purpose of this study, the following stance phase related characteristics of SO length were determined and analyzed for each cycle across 4 cats, for whom SO fascicle length was measured (Table 1), and experimental conditions: the MTU and fascicle lengthening in stance defined as the difference in normalized length between the initial length at PC and peak length in stance; and the MTU and fascicle shortening in stance defined as the difference in normalized length between the peak length in stance and the minimum length in terminal stance.

First, a single sample t-test was performed for each cat with implanted sonomicrometry crystals (Table 1) and slope to determine if SO fascicle shortening was present following denervation, i.e. was greater than zero (SPSS 20.0, SPSS Inc. Chicago, IL, USA). To determine effects of SO paralysis and walking slope condition on SO length characteristics, the linear mixed model analysis (SPSS 20.0, SPSS Inc. Chicago, IL, USA) was used to account for varying number of trials between the factors. For the linear mixed model analysis of individual cats, SO pre-post paralysis condition and walking slope were considered fixed factors, while the walking cycle was considered a random factor. When the mixed model analysis was applied to data from all cats pooled together, an additional random factor, cat, was introduced. The linear mixed model analysis was performed on each SO muscle length characteristic described above. The same analysis was also performed for separate sets of EMG data of SO, LG and MG muscles in 7 cats to test effects of denervation and walking slope on mean EMG burst activity. For one cat (CO), a single sample t-test was used to compare the absolute SO fascicle length at the end of stance against the minimum SO fascicle length during passive movement of the ankle joint. Finally, two-sample, unpaired t-tests were performed to compare the magnitude of change in SO fascicle shortening/lengthening post-denervation versus baseline between all walking conditions (for downslope (n = 25/27), level (n = 39/32) and upslope (n = 38/25), respectively; see Table 1). Statistical significance level was set at *P* *<* 0.05.

## Results

**Figure 1:**
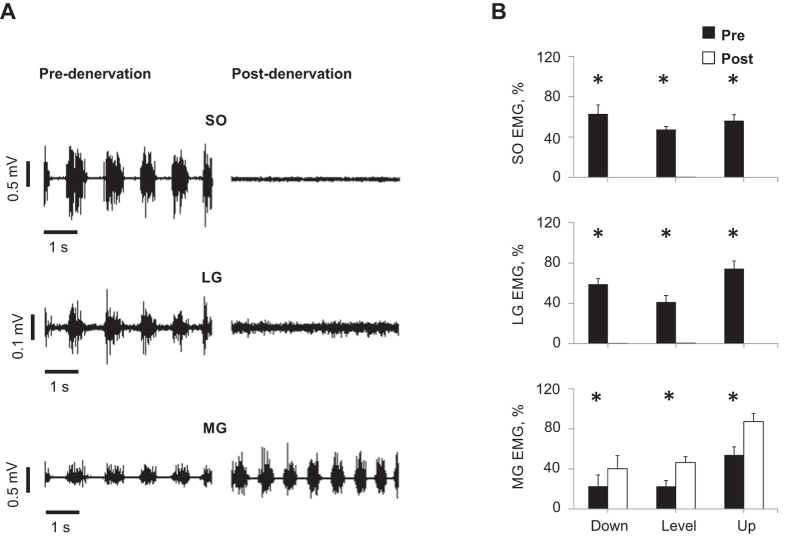
EMG activity of soleus (SO), lateral gastrocnemius (LG) and medial gastrocnemius (MG) muscles during level and slope walking before and after SO-LG denervation. (**A**) Representative raw EMG activity of SO, LG and MG before and after denervation for level walking; Cat CO. (**B**) Averaged normalized EMG activity (mean  ±  s.d.) of SO, LG and MG during downslope, level, and upslope walking before and after denervation; 7 cats (Table 1). *Significant differences between pre- and post-denervation (*P* < 0.001).

**Figure 2:**
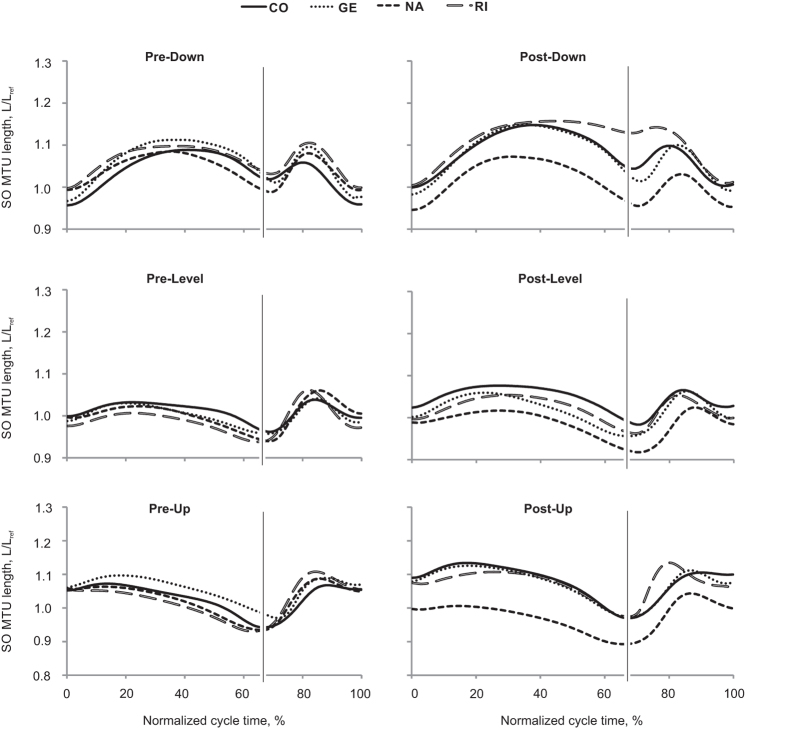
Normalized SO MTU length of each cat during three walking slope conditions, pre and post SO-LG denervation. Values are a fraction of the MTU reference length (L_ref_), which is the mean of the maximum and minimum MTU lengths during the swing phase of level walking before denervation. Time is normalized to the duration of stance phase and swing phase separately. The thin vertical line represents the end of stance phase and the beginning of swing phase. 4 cats: CO, GE, NA, RI (Table 1).

**Figure 3:**
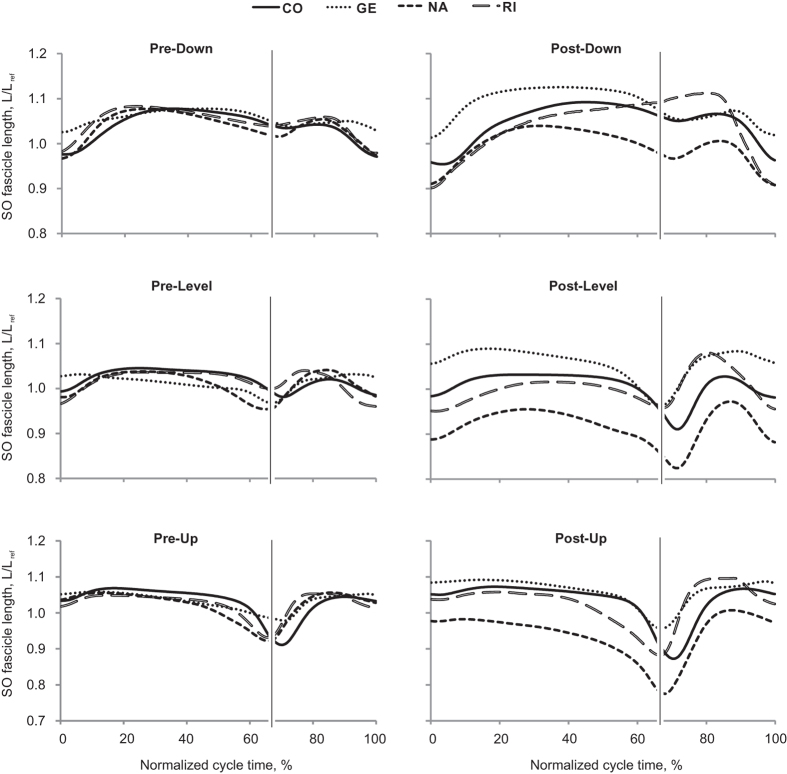
Normalized SO fascicle length of each cat during three walking slope conditions, pre and post SO-LG denervation. Values are a fraction of the fascicle reference length (L_ref_), which is the mean of the maximum and minimum fascicle lengths during the swing phase of level walking before denervation. Time is normalized to the duration of stance phase and swing phase separately. The thin vertical line represents the end of stance phase and the beginning of swing phase. 4 cats: CO, GE, NA, RI (Table 1).

**Figure 4:**
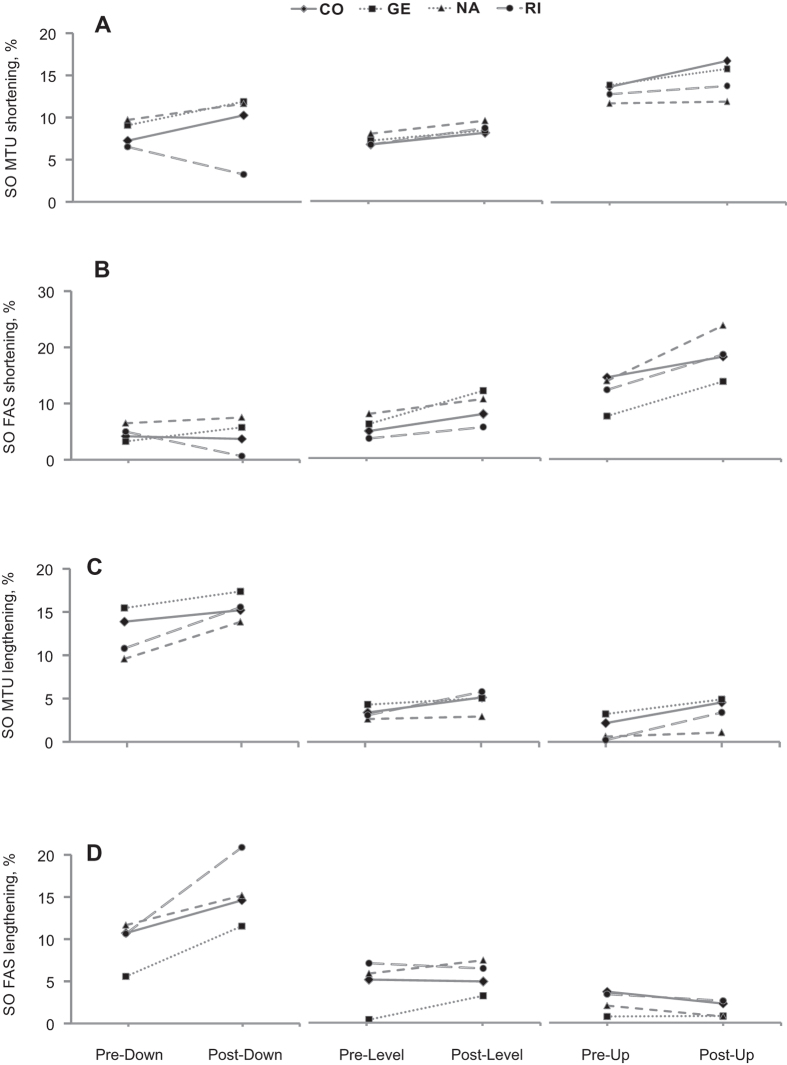
Normalized mean magnitude of shortening and lengthening of SO fascicles (FAS) and MTU during stance phase for each cat (CO, GE, NA, RI, Table 1) and walking slope condition pre and post SO-LG denervation. All values are a percentage of reference length Lref (see text for further explanations). (**A**) SO MTU shortening before and after denervation. (**B**) SO fascicle shortening pre and post denervation. (**C**) SO MTU lengthening before and after denervation. (**D**) SO fascicle lengthening pre and post denervation.

**Figure 5:**
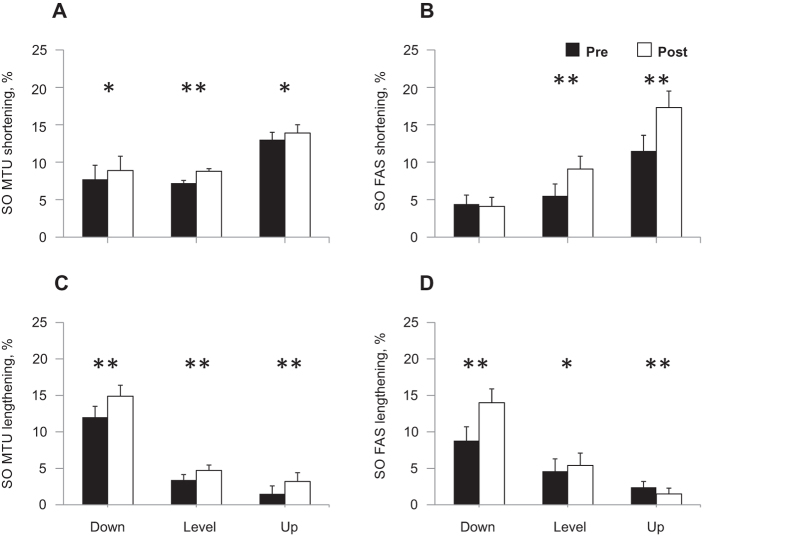
Normalized mean magnitude of shortening and lengthening of SO MTU and fascicles during stance of level and slope walking pre and post denervation averaged across 4 animals (CO, GE, NA, RI, Table 1). (**A**) Mean SO MTU shortening. (**B**) Mean SO fascicle (FAS) shortening. (**C**) Mean SO MTU lengthening. (**D**) Mean SO fascicle (FAS) lengthening. Values are a percentage of L_ref_ (mean ± s.d.). Significant differences between pre- and post-denervation values were found in all walking conditions except for SO FAS shortening during downslope walking. *P < 0.05, **P < 0.001.

During days 3 to 14 after SO-LG nerve transection and repair, SO and LG muscles were paralyzed, which was evident from the lack of EMG bursts during walking (Fig. 1). During the same period, the mean EMG burst magnitude in the intact MG muscle increased significantly (P < 0.05; Fig. 1). Although the general patterns of SO MTU length (Fig. 2) or fascicle length (Fig. 3) during level and slope walking were surprisingly similar pre- and post-nerve transection and repair, there were substantial differences in the magnitude of SO muscle shortening and lengthening during the stance phase of walking (Figs 4 and 5).

### Soleus MTU and fascicle shortening

SO MTU shortening during the stance phase increased significantly following denervation in all slope conditions (Fig. 5; mean ± s.d.: 7.7 ± 2.2 vs 8.9 ± 2.1%L_ref_, F_1,47_ = 4.45, *P* ≤ 0.001 for downslope walking; 7.2 ± 0.9 vs 8.8 ± 0.7%L_ref_, F_1,64_ = 15.23, *P* ≤ 0.001 for level; 13.0 ± 1.3 vs 13.9 ± 1.2%L_ref_, F_1,59_ = 4.74, *P* = 0.033 for upslope). Analysis of SO MTU shortening after SO denervation in individual animals revealed a significant increase in 2 out of 4 animals in each of the slope walking conditions (*P* ≤ 0.01), while one cat showed a decrease during downslope walking (*P* = 0.001, Fig. 4A). The change in SO MTU shortening in response to denervation differed only between level and upslope walking (1.6 ± 0.5 vs. 0.9 ± 1.5%L_ref_, respectively, *P* = 0.011). There were no significant differences between level and downslope (*P* = 0.393), and downslope and upslope (*P* = 0.595) conditions.

**Figure 6:**
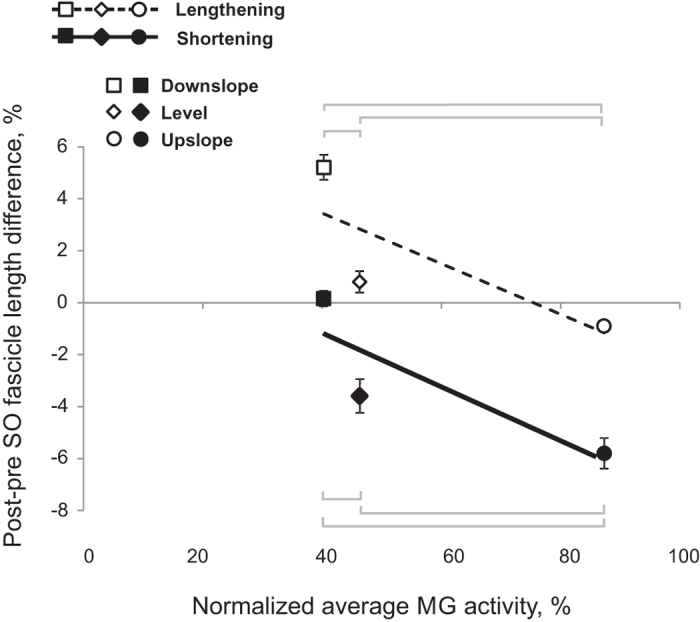
Mean post-pre difference in SO fascicle lengthening/shortening during stance as a function of mean MG activity post-denervation. The post-pre difference in fascicle shortening was computed as the difference in SO fascicle shortening between pre- and post-denervation values (see Fig. 5B); a negative value indicates the increase in shortening post denervation. The post-pre difference in fascicle lengthening was computed as the difference in SO fascicle lengthening between post- and pre-denervation values (Fig. 5D); a positive value means the increase in lengthening post denervation. Values are a percentage of L_ref_ (mean ± s.d.). Lines of best linear fit are shown for changes in SO fascicle shortening (solid line, black symbols) and SO fascicle lengthening (dashed line, open symbols). Squares, diamonds and circles correspond to downslope, level and upslope walking conditions, respectively. Significant differences between pairs of walking conditions are shown by horizontal brackets (*P* ≤ 0.001).

SO fascicle shortening persisted following denervation in all walking conditions. The majority of SO fascicle shortening occurred during the end of stance (approximately between 45% and 65% of cycle time, Fig. 3) before and after denervation. The magnitude of SO fascicle shortening during stance following denervation was significantly greater than zero in all cats and slope conditions (*P* ≤ 0.004; see Table 1 for number of samples) except for one cat in downslope condition (RI, *P* = 0.138, Fig. 4B). After denervation, SO fascicle shortening in stance increased on average by 65% and 56% in level and upslope walking, respectively. The corresponding mean normalized shortening magnitudes were 9.1 ± 1.7 and 17.3 ± 2.2%L_ref_, respectively, which were significantly greater than those before denervation (5.5 ± 0.2 and 11.1 ± 2.7%L_ref_; F_1,62_ = 50.04, *P* ≤ 0.001 and F_1,59_ = 61.33, *P* ≤ 0.001, respectively; Fig. 5B). The change in SO fascicle shortening in response to denervation was significantly greater for the upslope condition compared to the level condition (5.8 ± 3.0 vs. 3.6 ± 2.3%L_ref_, *P* ≤ 0.001, Fig. 6). Post denervation changes in fascicle shortening during upslope and level conditions were both greater than that during downslope condition (−0.3 ± 1.7%L_ref_; *P* ≤ 0.001; Fig. 6). The pre- versus post-denervated change in SO fascicle shortening for each walking condition was correlated to the corresponding mean MG EMG activity, with greater changes in fascicle shortening corresponding to higher EMG (Fig. 6, closed symbols).

### Soleus MTU and fascicle lengthening

There was a significant increase in SO MTU lengthening following denervation in all walking conditions (Fig. 5C). It increased by 22% for downslope walking (12.4 ± 2.5 vs. 15.1 ± 1.8%L_ref_, F_1,47_ = 34.648, *P* ≤ 0.001), 34% for level walking (3.5 ± 1.0 vs. 4.7 ± 1.9%L_ref_, F_1,62_ = 15.475, *P* ≤ 0.001), and by 200% for upslope walking (1.1 ± 1.2 vs. 3.3 ± 2.0%L_ref_, F_1,58_ = 35.739, *P* ≤ 0.001). SO MTU lengthening following denervation in individual cats increased in most walking conditions and cats (*P* ≤ 0.001–0.010; Fig. 4C); the exceptions were no changes in cat CO during downslope, GE during level and NA during level and upslope walking (*P* = 0.052–0.540). For the change in MTU lengthening in response to denervation, there was a significant difference between downslope and level walking (2.9 ± 2.1 vs. 1.3 ± 1.1%L_ref_, respectively, *P* ≤ 0.001), and downslope and upslope walking (2.9 ± 2.1 vs. 1.7 ± 1.6%L_ref_, *P* = 0.019). There was no difference between level and upslope walking (*P* = 0.234).

SO fascicle lengthening in stance increased after denervation in level and downslope walking by 17% and 59%, respectively (from 4.6 ± 2.9 to 5.4 ± 1.9%L_ref_, F_1,62_ = 7.845, *P* = 0.007 and from 8.8 ± 2.2 to 14.0 ± 2.9%L_ref_, F_1,46_ = 106.330, *P* ≤ 0.001, respectively); but decreased in upslope condition by 38% (from 2.4 ± 0.9 to 1.5 ± 0.8%L_ref_, F_1,58_ = 21.661, *P* ≤ 0.001; Fig. 5D). During downslope walking, fascicle lengthening increased after SO-LG denervation in three cats (*P* ≤ 0.001), but did not change in one cat (NA: F_1,7_ = 3.198, *P* = 0.117). During level walking, SO fascicle lengthening increased after denervation in only one cat from 0.5 ± 0.3 to 3.4 ± 1.3%L_ref_ (GE: F_1,19_ = 50.14, *P* ≤ 0.001, Fig. 4D). For the upslope condition, SO fascicle lengthening decreased significantly in two cats after denervation (NA: F_1,18_ = 42.34, *P* ≤ 0.001; CO: F_1,8_ = 79.04, *P* ≤ 0.001, respectively), whereas the remaining two cats showed no significant changes (RI: F_1,11_ = 0.946, *P* = 0.352, and GE: F_1,18_ = 0.305, *P* = 0.588). We found significant differences in the magnitude of change of SO fascicle lengthening in response to denervation between downslope, level, and upslope conditions (5.2 ± 2.7, 0.8 ± 2.4, and −0.9 ± 1.1%L_ref_, respectively; *P* ≤ 0.001; Fig. 6). There also appeared to be a close relationship between the change in SO fascicle lengthening and MG EMG activity after denervation (Fig. 6, open symbols).

### Fascicle lengths in passive and electrically stimulated SO muscle of sedated animal

SO fascicle length measured in one cat (CO) under sedation at a fully extended ankle (180^o^), corresponding to its shortest MTU length, was 35.7 mm. When the SO muscle was electrically stimulated via implanted EMG electrodes to cause maximum fascicle shortening at the same joint angle, SO fascicle length decreased to 32.7 mm. Following denervation, SO fascicle lengths of the same cat at the end of stance during downslope, level, and upslope walking were 38.7 ± 0.3 mm (the corresponding ankle joint angle was 103.9 ± 4.3^o^), 34.8 ± 0.6 mm (130.9 ± 2.6^o^), and 33.0 ± 0.5 mm (135.4  ±  4.8^o^), respectively. The fascicle lengths in the denervated SO during level and upslope walking were significantly shorter than the minimum fascicle length of passive SO in the sedated condition (*P* = 0.005 and *P* < 0.001, respectively). When compared to fascicle length of electrically stimulated SO in the sedated condition, fascicle lengths of the denervated SO at paw lift-off during level and downslope walking were longer (*P* < 0.001 and *P* < 0.001, respectively), whereas SO fascicle length during upslope walking was not different (one sample t-test, *P* = 0.308).

## Discussion

**Figure 7:**
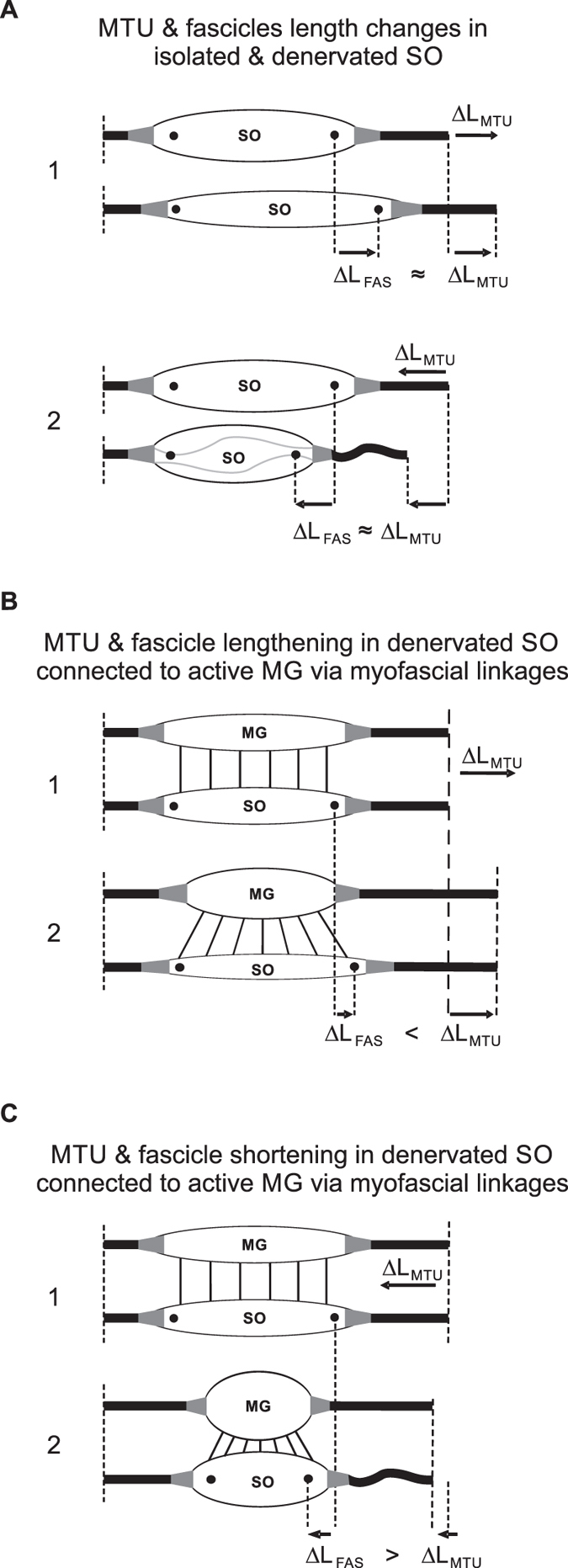
Schematic illustrating changes in SO fascicle length (ΔL_FAS_) during SO MTU length changes in different conditions. Black circles in SO indicate implanted sonomicrometry crystals to measure muscle fascicle length as distance between the crystals. (**A**) MTU and fascicle lengthening (1) and shortening (2) in passive SO muscle with no myofascial force transmission between SO and its synergists. During MTU lengthening, SO fascicles are expected to take up most of the MTU stretch. During MTU shortening at lengths below the SO MTU resting length (at which SO MTU produces no passive force), both the fascicles and tendon are expected to buckle (see text for further explanations). (**B**) MTU and fascicle lengthening in passive denervated SO while MG MTU is elongating starting from a passive condition (1) to a final elongation in which MG is active and its fascicles are shortened (2). In this situation, myofascial links between MG and SO fascicles might pull on SO muscle belly and reduce its elongation. (**C**) MTU and fascicle shortening in passive denervated SO while MG MTU is shortening starting from a passive condition (1) to a final shortening in which MG is active and its fascicles are shortened (2). In this situation, myofascial links between MG and SO fascicles might pool on SO fascicles and increase its shortening.

Previous studies have documented differential changes in MTU length and EMG of SO and MG in intact cats^19^,^36^,^38^, as well as muscle fascicle length changes in intact MG after denervation of SO and LG^13^ during level and slope walking. MTU length and EMG activity of SO and MG before denervation obtained in this study are in a good agreement with the previous studies. This report is the first to describe fascicle length changes in a denervated muscle during locomotion. We hypothesized (see Introduction) that given no myofascial force transmission between denervated SO and its intact synergists, SO fascicles would mirror lengthening of SO MTU during the stance phase of walking (Fig. 7A1), whereas during SO MTU shortening in the stance phase, denervated SO fascicles would not shorten substantially. The obtained results contradicted our hypothesis. They indicate that after denervation of SO and LG (i) there are differential changes in MTU and fascicle lengthening of SO in the yield phase of stance, i.e. during upslope walking, SO fascicle lengthening decreased while SO MTU lengthening increased (Fig. 5C,D); (ii) there is indeed SO fascicle shortening during the stance phase of walking; and (iii) the magnitude of the changes in shortening and lengthening appears to be related to the EMG magnitude of the intact synergist (MG, Figs 1B and 6) rather than to the SO MTU length changes (Fig. 5A,B), i.e. the increase in SO fascicle shortening and MG EMG activity change in parallel from downslope to level and to upslope walking conditions (Figs 1B,5B and 6). Finally, (iv) the fascicle length of the denervated SO at the end of fascicle shortening in stance during level and upslope walking was shorter than the passive SO fascicle length measured at a much more extended ankle angle and, thus, at a lower SO MTU length in one sedated animal.

### Passive Muscle Fascicle Elasticity and Fascicle Buckling

Any explanation for the observed behavior of SO fascicles would depend on assumptions about relative compliance of the tendon and muscle fascicles of the denervated (passive) SO MTU in the studied conditions. First, if the muscle fascicles were more compliant than the tendon, as shown to be the case in the cat SO at very small muscle forces, i.e. low EMG levels^22^,^39^, then lengthening of SO MTU would be mirrored in the muscle fascicles (Fig. 7A1). An increase in SO MTU lengthening in the yield phase of stance during all three slope conditions after denervation of the LG and SO muscles (Fig. 5C; see also^13^,^33^,^40^), would result in an increase in SO fascicle lengthening. In contrast, SO fascicle lengthening decreased during upslope walking following SO-LG denervation (Figs 5D and 6). This result, however, is consistent with the idea of increased external forces acting on the SO muscle belly and resisting its lengthening (see Fig. 7B2).

Second, shortening of the denervated SO MTU and muscle fascicles during the propulsion phase in stance occurs in most walking slope conditions (Figs 2, 3, 4, 5A,B). The fascicle lengths of the denervated SO at the end of the shortening phase during level and upslope walking were significantly shorter than the minimal possible fascicle length of passive SO measured in the same sedated animal at a much more extended ankle and thus at shorter SO MTU length (the latter condition is shown schematically in Fig. 7A2). Therefore, simple joint kinematics cannot explain the behavior observed in the denervated SO fascicles during walking. Also, shortening of muscle fascicles of denervated SO by its passive forces in this situation does not seem likely. This is because passive forces of cat SO within the *in vivo* range of motion at the ankle joint (above 90^o^–120^o^, see Introduction) are very small (peak passive forces do not exceed 0–10% of SO maximum isometric force or peak SO tendon force during walking^20^,^21^,^41^,^42^. In addition, buckling of passive SO fascicles^25^,^26^ (see Fig. 7A2) during SO MTU shortening in the stance phase of walking (Figs 2 and 5A) appears unlikely due to high pressure inside the superficial posterior crural compartment^27^,^28^,^43^ caused by actively contracting intact ankle extensor synergists, such as MG (Fig. 1)^13^ and plantaris^14^. The above arguments suggest that length changes of SO fascicles during stance cannot be explained solely by SO intrinsic passive forces or fascicle buckling. Therefore, other mechanisms must contribute to the observed behavior.

### Epimuscular Myofascial Force Transmission

Our results support the presence of epimuscular myofascial force transmission between the denervated SO and intact ankle extensors based on three key findings; (1) lengthening of denervated SO fascicles during the yield phase in stance does not correspond to SO MTU lengthening (compare upslope walking in Fig. 5C,D) and appears to decrease with increasing synergist muscle activity (Figs 1B; see also Fig. 7A1), (2) passive SO fascicles shorten during the push-off phase in late stance (Figs 3 and 5B), despite the expected high pressure inside the superficial posterior crural compartment preventing buckling of passive SO fascicles (see discussion above and Fig. 7A2), and (3) SO fascicle shortening following denervation appears to increase with levels of synergist muscle activity (MG, Figs 1B and 6; plantaris^14^) during level and slope walking conditions (compare Figs 1B and 5B).

Epimuscular myofascial force transmission is mediated by connective tissue structures at the muscle belly boundaries. In normal physiological conditions myofascial force transmission between SO and synergistic ankle extensor muscles in cats and rats was found to be limited^23^,^24^. Denervation of SO results in an increased activity of the intact synergistic MG during walking (Fig. 1 and references^13^,^14^). In addition, SO-LG denervation leads to a greater yield at the ankle and a smaller knee flexion during the stance phase of walking^12^,^33^. As a consequence, the MTU length of the denervated SO and intact MG muscles increases following SO-LG denervation and this increase is larger for MG than SO. This in turn might result in a higher than normal relative displacement between SO and MG muscle bellies. It has been shown that the extent of myofascial force transmission is dependent on this relative position^16^,^17^,^44^,^45^, as this affects the length and stiffness of the epimuscular linkages. We propose that following SO-LG denervation, forces exerted by these linkages on the denervated SO may cause reduced lengthening (during the yield phase) or increased shortening (during push-off phase) of SO muscle fascicles. An illustration of this proposed mechanism is presented in Fig. 7B,C, respectively. Such an effect of epimuscular connective tissues is in agreement with its previously hypothesized function as a safety net for traumatic events in muscles and tendons^46^.

### Potential Functional Implications of Increased Fascicle Length Changes in Denervated Muscles

Our findings may have several functional implications. The presence of unexpectedly large length changes in the paralyzed muscle as a result of peripheral nerve injury could result in decreased muscle atrophy^7^,^8^. Several studies have reported that repetitive lengthening of paralyzed muscles reduces the effect of disuse on muscle atrophy through stretch- and/or stress-induced activation of signaling pathways responsible for muscle growth^47^,^48^,^49^,^50^,^51^. Therefore, passive lengthening and shortening of muscle fascicles in denervated muscles, as observed in this study, may provide a mechanism by which atrophy of paralyzed muscles may be reduced.

Paralysis or reduction in force output (e.g., due to atrophy) of a single muscle in a muscle group may lead to asymmetric loading of the joint and subsequent development of joint tissue degeneration, osteoarthritis, and possible other secondary joint conditions^9^,^10^,^11^. Fascicle shortening and tendon lengthening within a paralyzed muscle may contribute to maintaining a more symmetric loading pattern in the joint by redistributing forces to tendons of all synergists in the group, including those of injured muscles. A reduction in muscle atrophy, as mentioned in the paragraph above, would also help to reduce any asymmetry after muscle function is restored.

### Future Studies

This study was designed to document fascicle length changes in denervated SO during locomotion. Although the suggested mechanism of myofascial force transmission between denervated SO and its intact synergists (e.g. MG) appears to be the most probable explanation of our unexpected findings (see discussion above), direct testing is required to prove this. One approach could be to sever the myofascial linkages between SO and MG. This, however, will not likely result in the desired condition. Due to extreme plasticity of connective tissues, which tend to quickly (within a few days) reestablish original or grow new connections^52^,^53^,^54^, it will be very difficult to obtain measurements in which full isolation of these muscles can be assured. Another approach could be to attenuate effects of myofascial linkages by injecting type A botulinum toxin into the muscle as was recently reported^55^. However, such an intervention will affect the force producing capacity in both the target as well as adjacent muscles^56^. Probably the most adequate approach will be to sequentially denervate the synergists.

## Conclusion

We have presented the first report of increased muscle fascicle length changes in denervated SO fascicles during the stance phase of walking that might potentially affect the extent of muscle atrophy and symmetry of joint loading. Evidence from SO fascicle and MTU length, as well as from MG EMG activity recordings suggests that this behavior might result from the action of epimuscular myofascial linkages between intact and paralyzed ankle extensors. Additional studies are needed to confirm the importance of this mechanism.

## Additional Information

**How to cite this article**: Mehta, R. *et al.* Unexpected Fascicle Length Changes in Denervated Feline Soleus Muscle During Stance Phase Of Walking. *Sci. Rep.* **5**, 17619; doi: 10.1038/srep17619 (2015).

## Figures and Tables

**Table 1:** Number of walking cycles from each cat and slope condition analyzed before and after denervation.

Slope	Cat	SO Length		SO EMG		LG EMG		MG EMG	
		Pre	Post	Pre	Post	Pre	Post	Pre	Post
Level	GE	11	10	10	13	10	13	10	13
	CO	8	7	8	10	4	10	8	10
	NA	8	10	–	–	–	–	–	–
	RI	5	12	–	–	–	–	–	–
	IN	–	–	10	10	14	10	–	–
	KO	–	–	6	10	–	10	–	10
	BL	–	–	12	8	–	8	–	8
	Total	32	39	46	51	38	51	18	41
Up	GE	4	16	10	10	5	10	10	6
	CO	5	5	5	9	10	9	5	9
	NA	8	12	2	6	–	6	–	6
	RI	8	5	–	–	–	–	–	–
	IN	–	–	10	10	11	10	–	–
	KO	–	–	10	10	10	10	10	10
	BL	–	–	10	6	–	6	10	6
	Total	25	38	47	51	36	51	35	37
Down	GE	5	10	13	12	11	12	10	10
	CO	12	4	1	4	1	4	–	–
	NA	3	7	4	5	4	5	–	–
	RI	7	4	–	–	–	–	–	–
	IN	–	–	10	8	10	8	–	–
	KO	–	–	9	11	2	11	3	11
	BL	–	–	11	6	–	6	–	3
	Total	27	25	48	46	28	46	13	24

SO length variables include fascicle lengthening/shortening and MTU lengthening/shortening.
